# Severe colitis after PD-1 blockade with nivolumab in advanced melanoma patients: potential role of Th1-dominant immune response in immune-related adverse events: two case reports

**DOI:** 10.1186/s12885-019-6138-7

**Published:** 2019-10-29

**Authors:** Koji Yoshino, Takayuki Nakayama, Ayumu Ito, Eiichi Sato, Shigehisa Kitano

**Affiliations:** 1 Department of Dermato Oncology, Tokyo Metropolitan Cancer and Infectious Disease Center Komagome Hospital, 3-18-22 Honkomagome, Bunkyo-ku, Tokyo, 113-8677 Japan; 2 Department of Experimental Therapeutics, National Cancer Center Hospital, Tokyo, Japan; 3 Department of Hematopoietic Stem Cell Transplantation, National Cancer Center Hospital, Tokyo, Japan; 4 Department of Pathology, Institute of Medical Science, Medical Research Center, Tokyo Medical University, Tokyo, Japan; 5 Division of Cancer Immunotherapy, Exploratory Oncology Research and Clinical Trial Center, National Cancer Center, 5-1-1 Tsukiji, Chuo-ku, Tokyo, 104-0045 Japan

**Keywords:** Autoimmune colitis, Nivolumab, Immune-related adverse event, Biomarker, C-reactive protein, Case report

## Abstract

**Background:**

Nivolumab is an immune checkpoint inhibitor specific to the programmed death 1 (PD-1) receptor. Nivolumab has shown clinical responses in many malignancies. Although immune-related adverse events (irAEs) associated with nivolumab are largely tolerable, severe irAEs have occurred in some patients. However, the mechanisms underlying the development of irAEs are not fully clarified.

**Case presentation:**

We report 2 patients with metastatic melanoma who developed colitis, an irAEs caused by nivolumab. Both patients experienced colitis after nivolumab administration. Pathological examination of the colon showed robust infiltration of CD8^+^ cells and T-bet expressing CD4^+^ cells in both cases, indicating helper T cells (Th) 1 to be responsible for the dominant response. Additionally, we observed the serum C-reactive protein level (CRP) as well as interleukin-6 (IL-6) reflected the clinical course of irAEs clearly in the two cases.

**Conclusion:**

Our two cases suggested that the development of irAEs due to nivolumab is associated with Th1 dominant response. CRP as well as IL-6 was found to be a potential biomarker for irAEs. Our findings may help to understand the mechanisms underlying irAEs caused by nivolumab and manage irAEs in clinical practice.

## Background

The advent of immune checkpoint inhibitor development has offered clinical benefits in a variety of malignancies including melanoma. Nivolumab is a fully humanized monoclonal IgG4 antibody directed against programmed cell death 1 (PD-1), which is expressed on activated T cells and functions as a co-inhibitory receptor. Despite their encouraging efficacies, however, immune checkpoint inhibitors carry risks of treatment-related complications associated with harmful autoimmune responses, which are referred to as immune-related adverse events (irAEs). While the safety profile of nivolumab monotherapy is generally acceptable, with common adverse toxicities including fatigue, rash, pruritus, and diarrhea, there are reports of patients requiring treatment interruption and corticosteroid administration [1, 2]. In a previous phase II clinical trial, severe irAEs (grade 3/4 according to NCI CTCAE guidelines) occurred in 16.3% of treated patients [3]. Although colitis is the most common irAE in patients treated with anti-cytotoxic T-lymphocyte antigen 4 (CTLA-4) antibodies, the rate of grade 3/4 diarrhea in those given PD-1/ programmed cell death ligand 1 (PD-L1) agents is very low (1 to 2%) [4–6]. However, autoimmune colitis can be severe with potentially fatal perforations [7]. Although irAEs associated with nivolumab have gradually been recognized, the mechanisms underlying these irAEs have not as yet been fully clarified. Herein, we report 2 melanoma patients who developed severe colitis during nivolumab treatment and whose pathological findings of colon we could compare between before and after corticosteroid treatment. We analyzed biological samples from the patients and discuss, with a review of the literature, the pathophysiology of this complication.

## Case presentation

### Case 1

**Fig. 1:**
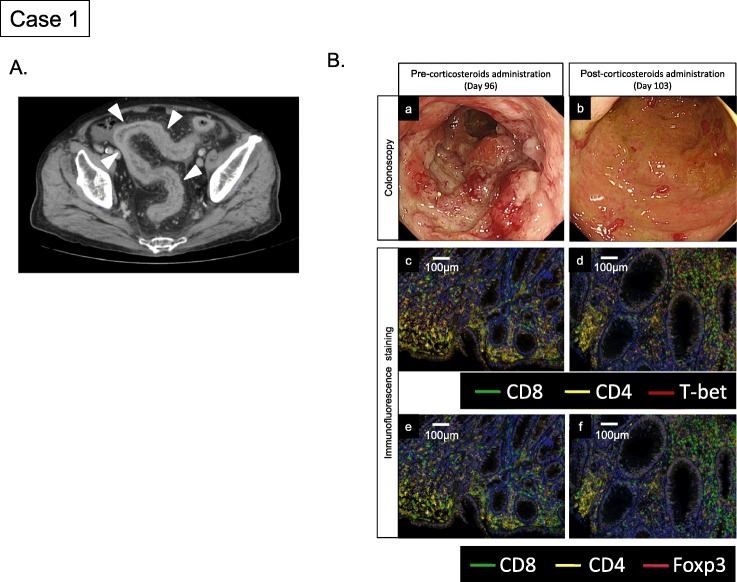
Clinicopathological findings of colitis in case 1: **a** Computed tomography of the abdomen on Day 95 after initiation of nivolumab showed an edematous lesion involving the rectosigmoid colon. **b** (a) Colonoscopy on Day 96 revealed an ulcerative lesion in the rectosigmoid colon. (b) Colonoscopy on Day 103 revealed that ulcerative lesion was improved but remained after corticosteroids administration. (c) (d) Immunohistochemical staining (CD8, CD4 and T-bet) of colon biopsy samples on Day 96 and Day 103. (e) (f) Immunohistochemical staining (CD8, CD4 and Foxp3) of colon biopsy samples on Day 96 and Day 103 showed increased infiltration of CD8^+^ and Foxp3^+^ cells after corticosteroids administration

The patient was an 80-year-old man with malignant melanoma of the neck. His medical history included diabetes and ischemic heart disease, but no autoimmune diseases. At diagnosis, his performance status (PS) was 1. The primary tumor was a 2.4-mm-thick lesion with no ulceration, and BRAF mutations were negative. No obvious metastatic lesions were detected clinically. The primary tumor was resected with lymph node dissection, identifying micro-metastasis in one sentinel node. The pathologic stage was IIIB (pT3a, N2a, M0 by TNM classification). He received 5 cycles of adjuvant therapy with interferon beta at a dose of 3-million units per body every 7 weeks. After a 6-month treatment-free period, follow-up computed tomography (CT) revealed a metastatic lesion in the lung. Then, at 1 year after the original diagnosis, nivolumab treatment was started at a dose of 2 mg/kg every 3 weeks. On day 64, after 4 administrations of nivolumab, the patient presented with mild diarrhea. On day 92, upon returning to our institution for the fifth nivolumab administration, he showed intractable diarrhea, a fever of 39 °C, and fatigue. He complained of passing watery and bloody stools more than 12 times per day. Nivolumab was discontinued and he was hospitalized to undergo intensive examinations and treatment. Abdominal CT showed intestinal edema, suggesting severe mucosal inflammation (Fig. 1a). Anti-bacterial treatment was immediately started with ampicillin-sulbactam (6 g/day). The fecal examination showed no signs of infectious bacteria. Colonoscopy revealed ulcerative lesions (full-circumference mucosal defect), especially in the sigmoid colon and more distal segments (Fig. 1B [a]). To assess the microenvironment of those lesions, multiplexed fluorescent immunohistochemistry was conducted in the same way as that performed in our study [8]. Immunohistochemical analysis of the colon biopsy showed severely inflamed mucosa with infiltration of CD8^+^ cells and T-bet expressing CD4^+^ cells (Fig. 1B [c]). T-bet expressed in both CD4^+^ and CD8^+^ cells. Infiltration of GATA3^+^ and RORγt^+^ cells were not obvious (Additional file 1 Figure S1). Based on these findings, administration of corticosteroids (0.5 mg/kg ≒ 30 mg/body) was started with a diagnosis with autoimmune colitis. However, the diarrhea was not fully recovered. One week after corticosteroids administration, the colonoscopy showed ulcerous lesion was improved but remained (Fig. 1B [b]). The colon biopsy samples revealed residual infiltration of CD8^+^ cells (Fig. 1B [d]). In addition, infiltration of Foxp3^+^ cells was more prominent than before corticosteroids administration (Fig. 1B [e] and [f]). On day 106, the corticosteroid dose was increased to 60 mg/body due to the persistent diarrhea, with passage of mucous and bloody stools more than 10 times per day. On day 108, based on the inflammatory laboratory findings having subsided, corticosteroid tapering was started and the antibiotic was stopped. At that time, the disease status was evaluated as a partial response. On day 113, the diarrhea showed gradual improvement, and dietary intake was restarted. By day 134, the corticosteroid dose was decreased to 5 mg/body and the diarrhea showed complete resolution. On day 144, based on overall improvement, we planned to discharge this patient. However, he developed a fever of 40 °C. Blood culture detected Klebsiella pneumonia, suggesting bacterial translocation from the intestines. Sepsis was complicated by disseminated intravascular coagulation and acute respiratory distress syndrome. On day 152, the patient died of multiple organ failure.

### Case 2

**Fig. 2:**
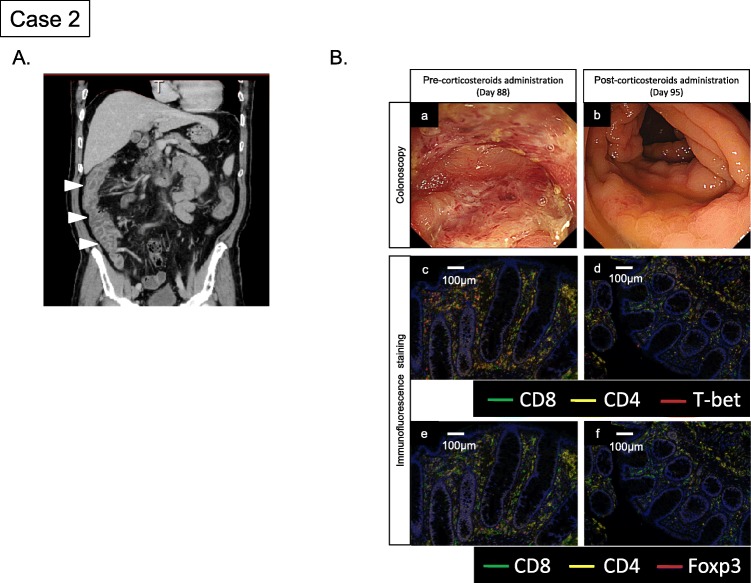
Clinicopathological findings of colitis in case 2: **a** Computed tomography of the abdomen on Day 87 after initiation of nivolumab showed an edematous lesion involving the rectosigmoid colon. **b** (a) Colonoscopy on Day 88 revealed erosion and an ulcerative lesion from the cecum to the sigmoid colon. (b) Colonoscopy on Day 95 showed improvement of colitis after corticosteroids administration. (c) (d) Immunohistochemical staining (CD8, CD4 and T-bet) of colon biopsy samples on Day 88 and Day 95. (e) (f) Immunohistochemical staining (CD8, CD4 and Foxp3) of colon biopsy samples on Day 88 and Day 95 showed reduced infiltration of CD8^+^ and T-bet^+^ cells after corticosteroids administration

The second case was a 58-year-old man with malignant melanoma involving the nail plate of the right middle finger. He had a past history of arrhythmia, but no autoimmune diseases. At the time of diagnosis, the primary lesion was 1.2 mm in thickness and clinically localized. The patient underwent curative surgical treatment. His melanoma harbored no BRAF mutations. The pathologic stage was IB (pT2a, N0, M0 by TNM classification). At 4 years after the initial diagnosis, a follow-up examination detected multiple bone metastases in the spine and ribs. He received radiotherapy for the spinal lesions (30 Gy in 10 fractions). Nivolumab was then started at the same dosing schedule as that used for case 1 (2 mg/kg every 3 weeks). The treatment was initially well tolerated. On day 87, after 4 administrations of nivolumab, the patient complained of watery diarrhea and mild abdominal distension. Abdominal CT showed a thickened intestinal tract, and colonoscopy revealed edematous and inflamed mucosa (Fig. 2A and B [a]). The pathological examination of the colon showed marked infiltration of CD8^+^ cells and T-bet expressing CD4^+^ cells, similar to the findings in case 1 (Fig. 2B [c]). Infiltration of GATA3^+^ and RORγt^+^ cells were not prominent (Additional file 1: Figure S1). Case 2 patient was hospitalized and the nivolumab treatment was temporarily interrupted. In this patient, colitis as an irAE was strongly suspected, prompting immediate administration of corticosteroids (60 mg/body ≒ 1 mg/kg). The fecal examination showed no signs of infectious enteritis. The diarrhea resolved rapidly in response to the corticosteroids and dietary intake was restarted. Colonoscopy findings was also improved a week after corticosteroids administration and we found CD8^+^ and T-bet^+^ cells were reduced from the findings of colon biopsy samples (Fig. 2B [b] and [d]). Infiltration of Foxp3^+^ cells was also reduced after corticosteroid administration (Fig. 2B [e] and [f]). The patient was then discharged and nivolumab was restarted. On day 141, after 6 administrations of nivolumab, he developed a fever of 38 °C. Although he had no signs of either obvious dyspnea or hypoxemia, chest X-ray revealed bilateral pneumonia. He was hospitalized and nivolumab treatment was again interrupted. Chest CT showed consolidation with air bronchograms in the left lower lobes (S8 and S9), and scattered consolidation in the right lower lobes. Bronchoscopic examination detected no apparent abnormalities, including purulent discharge. Pulmonary irAE (pneumonitis) was suspected and a 60 mg/body dose of corticosteroids was started. After 1 week, steroid tapering was initiated (10 mg/body/week to 30 mg, and thereafter 5 mg/body/week until cessation). However, the patient experienced 2 recurrences of pulmonary irAE during the steroid tapering period and was administered 60 mg/body doses of corticosteroids with subsequent amelioration of this condition. After resolution of the pulmonary irAE, the patient was treated with ipilimumab and re-treated with nivolumab. However, the melanoma had continued to progress and the patient died of melanoma progression on day 355.

### Additional analysis

**Fig. 3:**
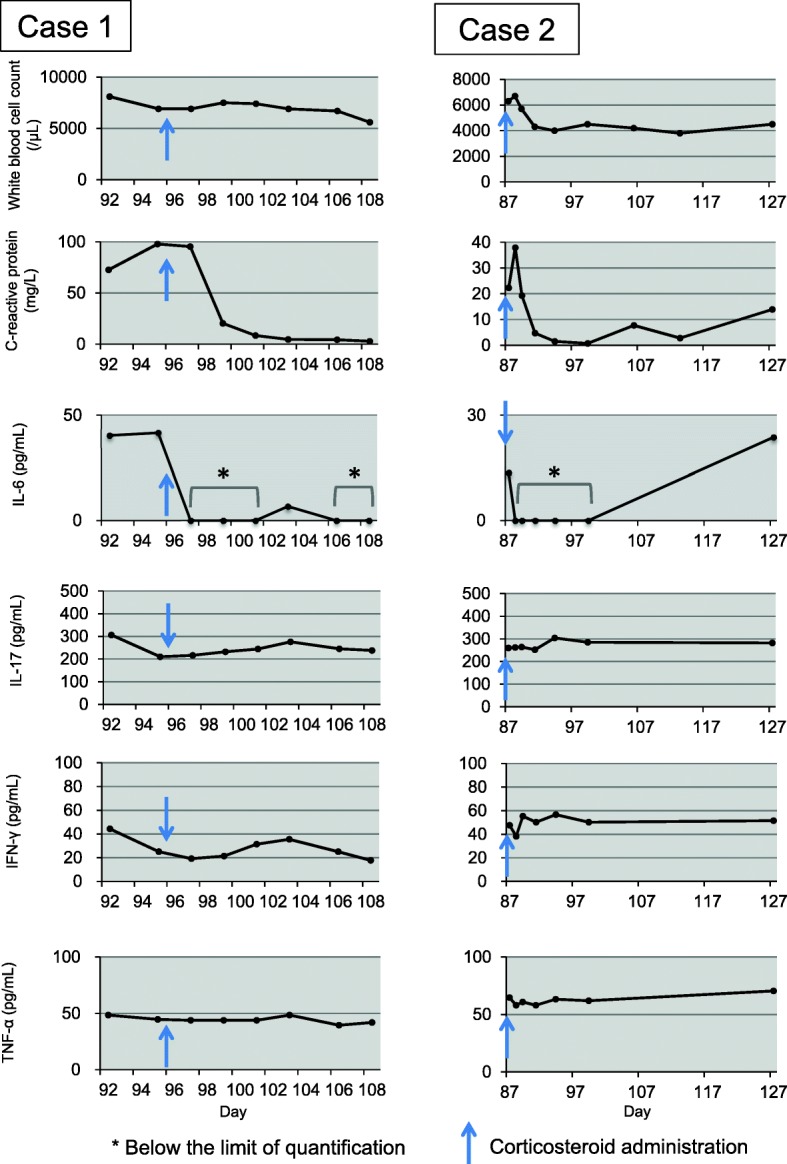
Temporal changes in white blood cell counts, serum C-reactive protein levels, and blood cytokine levels (IL-6, IL-17, IFN-γ, and TNF-α)

Stored blood samples were retrospectively examined to analyze for the temporal changes in cytokine levels (Fig. 3). The commercially available cytokine panel (Bio-Plex ProTM Human Cytokine 27-plex Assay; Bio-Rad Laboratories) was used for this analysis in accordance with the manufacturer’s instructions. We found that the serum C-reactive protein (CRP) levels and interleukin-6 (IL-6) levels were decreased after corticosteroid administration in both cases (Fig. 3 and Additional file 2: Table S1). The decrease in CRP and IL-6 was proportionate to the clinical resolution of the colitis.

## Discussion and conclusions

In the two cases reported herein, nivolumab caused colitis with marked infiltration of CD8^+^ cells and T-bet expressing CD4^+^ T cells, indicating helper T cells (Th) 1 to be responsible for the dominant response. To the best of our knowledge, no previous reports have described Th1-dominant response was associated with irAEs caused by nivolumab. In addition, serum CRP levels and IL-6 levels were proportionate to the severity of colitis apparently caused by nivolumab more clearly than other laboratory data and serum cytokines.

Accumulating evidence suggests that immune checkpoint inhibitors confer a survival benefit in patients with several types of cancer, including melanoma. With the extension of indications for immune checkpoint inhibitors, the recognition of serious irAEs becomes more and more vital for the safe use of these agents. Although the frequency and severity of irAEs following nivolumab monotherapy appear to be lower than those associated with CTLA-4 blockade, only limited information is currently available as to the diagnosis and management of, and the risk factors for, irAEs during nivolumab monotherapy [9]. Although it is generally accepted that most irAEs arise from immune activation, the mechanistic details are poorly understood [10]. Several studies have reported immunological analyses of colitis caused by immune checkpoint inhibitors. Immunohistochemical analysis of colon biopsy specimens from anti-CTLA-4 antibody recipients who developed colitis showed no evidence of Foxp3^+^ regulatory T cell depletion [11]. In contrast, however, another study revealed a marked increase in all T-cell subsets (CD3^+^, CD4^+^, and CD8^+^ cells) and of CD4^+^CD25^+^ regulatory T cells in patients with gastroenteritis due to anti-CTLA-4 antibody [12]. Ulcerative colitis and Crohn’s disease, which are the two most common inflammatory bowel diseases (IBD), are thought to have different pathogeneses [13]. Small bowel inflammation in Crohn’s disease is generally associated with increased IFNγ and IL17A expressions (indicative of Th1 and T helper 17 [Th17] cells, respectively), whereas type 2 T helper (Th2) cytokines (e.g., IL-4, IL-5, IL-13) predominate in ulcerative colitis [14]. Our studies of tissue specimens from these two cases suggested marked infiltrations of CD8^+^ cells and T-bet^+^ cells in CD4^+^ cell as well as CD8^+^ cells, indicating the Th1 dominant response to be a causative factor in irAEs associated with nivolumab. Although there are some differences between etiology of colitis induced by nivolumab and that of IBD, both diseases share some clinical findings [15, 16]. Previous report showed that both diseases have similarities in endoscopic and histopathological findings [15]. Interestingly, pathophysiology of both diseases is thought to be associated with gut microbiome [16]. Immune related colitis induced by nivolumab could be managed by treatment similar to that for IBD [15]. To study the similarities and differences between colitis induced by nivolumab and ulcerative colitis will lead to elucidate the pathophysiology of the both diseases. Therefore, it might be worth doing a comparative study of both diseases by immunoprofiling using multiplex IHC.

Tumor-infiltrating CD8^+^ lymphocytes appear to be a favorable prognostic factor in the vast majority of cancers [17]. On the other hand, previous study showed that the subepithelial layer was enriched with CD8^+^ T cells in colitis induced by anti-PD-1 antibodies, whereas CD4^+^ T cells were predominant in colitis associated with anti-CTLA-4 antibodies [18]. The cytotoxic T lymphocyte expressing CD8 may play a major role in both the efficacy of and the adverse events caused by nivolumab. However, the precise roles of Th cells in irAEs remain unknown. The results of our study suggest that Th1 cells have a considerable impact on irAEs. In addition, infiltration of Foxp3^+^ regulatory T cells was prominent in case 1 whose colitis persisted even after corticosteroid administration. Activated human CD4^+^ and CD8^+^ T cells transiently express Foxp3 [19]. In our 2 cases, however, Foxp3 expression was almost independent of T-bet and CD8 expression according to immunohistochemical findings, indicating most Foxp3^+^ cells reflected regulatory T cells. Those findings suggest that regulatory T cells might play a role in the recovery from colitis due to nivolumab. However, our findings are based on analysis of only two patients. Thus, further study is needed to confirm our findings.

Biomarkers possibly predicting the development of toxicities have been explored in patients receiving immune checkpoint inhibitor treatment. An increase from baseline in IL-17 after treatment was shown to be associated with irAEs [20]. IL-17 is one of the central inflammatory cytokines upregulated in the inflammatory bowel diseases [21]. In our study, although cytokines were not measured at baseline, specific biomarkers including IL-17 that reflect the severity of symptoms or pharmacological responses to nivolumab were not identified. The pathological findings also revealed mild infiltration of Th17 cells (RORγt^+^ cells) into the colon, indicating that Th17 cells may only play relatively minor roles in irAEs. On the other hand, in our 2 cases, CRP and IL-6 elevations were blunted by corticosteroid administration, in parallel with the resolution of colitis. CRP is an acute phase protein synthesized by the liver that serves as an early marker of inflammation or infection. The synthesis of CRP is stimulated by IL-6 and levels of CRP are strongly correlated with serum levels of IL-6. Although it is difficult to examine IL-6 routinely in daily clinical practice, assays for measuring CRP are available in routine clinical practice and are inexpensive. Several reports have suggested CRP to be a potential biomarker for autoimmune disorders including inflammatory bowel diseases [22, 23]. Previous study showed that IL-6 level in melanoma patients was elevated compared to healthy donor and tended to increase in patients with advanced stage [24]. The clinical courses and findings of our cases suggest that CRP as well as IL-6 reflects the treatment responses of irAEs caused by immune checkpoint inhibitors. Although many reports suggested CRP reflect the disease state in various cancers including melanoma [25, 26], examination of CRP kinetics could help for treatment of irAE in cancer patients. In addition, CRP is easier to measure than IL-6 in clinical setting. Thus, routine measurement of CRP may facilitate the prediction of the clinical course of irAEs. However, it should be noted that CRP could be affected by several clinical factors such as malignancies, infection, and administration of corticosteroid. Baseline CRP level was diverse especially in patients with malignancies. CRP levels were elevated (≥ 10 mg/dL) in 10.7% of melanoma patients at baseline [27]. Therefore, when we confirm recovery from irAEs, it is thought to be important to check whether decrease of CRP is in parallel with improvement of the other factors.

In conclusion, the cases presented herein suggested robust infiltration of CD8^+^ cells and T-bet^+^ cells in CD4^+^ as well as CD8^+^ T cells, indicating a Th1 dominant response, to be associated with the mechanism underlying the development of irAEs due to nivolumab. Additionally, CRP as well as IL-6 was found to be a potential biomarker reflecting treatment responses in patients with irAEs. Further pathological studies are needed to enrich our understanding of irAEs.

## Supplementary information

**Additional file 1: Figure S1.** Immunohistochemical staining of colon biopsy samples: A. Case 1. B. Case 2. Description of data: Infiltration of CD8+ and T-bet+ cells were marked in both cases whereas GATA3+ and RORγt+ cells were not obvious in both cases.

**Additional file 2: Table S1.** Temporal trends of blood cytokine levels (pg/mL) other than those listed in Fig. 3: A. Case1, B. Case2.

## Data Availability

The dataset supporting the conclusion of this article is owned by Tokyo Metropolitan Cancer and Infectious Disease Center Komagome Hospital but could be made available on request. Personal information will not be provided to ensure anonymity of the patient.

## Abbreviations

CT: Computed tomography
CTLA-4: Cytotoxic T-lymphocyte antigen 4
irAEs: Immune-related adverse events
PD-1: Programmed death 1
PD-L1: Programmed cell Death ligand 1
Th: Helper T cells
