# Supplementary material for: Severe colitis after PD-1 blockade with nivolumab in advanced melanoma patients: potential role of Th1-dominant immune response in immune-related adverse events: two case reports
Source: BMC Cancer. 2019 Oct 29;19:1019. doi: 10.1186/s12885-019-6138-7 (PMC6819390; doi:10.1186/s12885-019-6138-7)
Supplement: Supplementary file 1 — Additional file 1: Figure S1. Immunohistochemical staining of colon biopsy samples: A. Case 1. B. Case 2. Description of data: Infiltration of CD8+ and T-bet+ cells were marked in both cases whereas GATA3+ and RORγt+ cells were not obvious in both cases. [file 12885_2019_6138_MOESM1_ESM.pptx]

## Slide 1
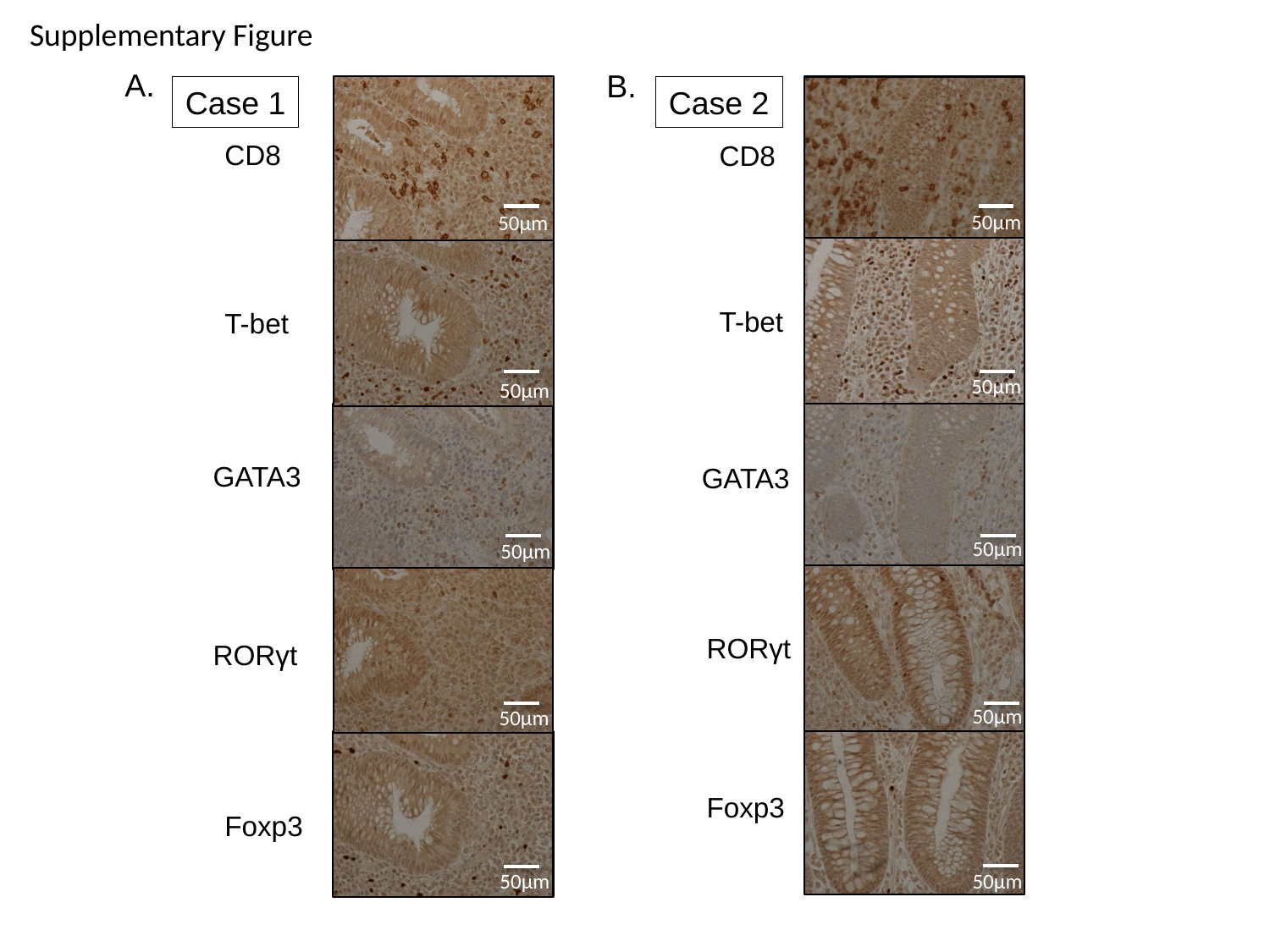

Supplementary Figure
A.
B.
Case 1
Case 2
CD8
CD8
50μm
50μm
T-bet
T-bet
50μm
50μm
GATA3
GATA3
50μm
50μm
RORγt
RORγt
50μm
50μm
Foxp3
Foxp3
50μm
50μm
