# Supplementary material for: Severe colitis after PD-1 blockade with nivolumab in advanced melanoma patients: potential role of Th1-dominant immune response in immune-related adverse events: two case reports
Source: BMC Cancer. 2019 Oct 29;19:1019. doi: 10.1186/s12885-019-6138-7 (PMC6819390; doi:10.1186/s12885-019-6138-7)
Supplement: Supplementary file 2 — Additional file 2: Table S1. Temporal trends of blood cytokine levels (pg/mL) other than those listed in Fig. 3: A. Case1, B. Case2. [file 12885_2019_6138_MOESM2_ESM.docx]

Supplementary table

A.

| Day | 92 | 95 | 96 | 97 | 99 | 101 | 103 | 106 | 108 |
| --- | --- | --- | --- | --- | --- | --- | --- | --- | --- |
| Event |  |  | corticosteroid administration |  |  |  |  |  |  |
| IL-1β | 6.74 | 1.69 |  | BLQ | 1.33 | 1.51 | 1.57 | 1.35 | BLQ |
| IL-1RA | 356 | 155 |  | 103 | 68.1 | 81.7 | 95.6 | 96.4 | 72.1 |
| IL-2 | BLQ | BLQ |  | BLQ | BLQ | BLQ | BLQ | BLQ | BLQ. |
| IL-4 | 5.38 | 4.48 |  | 4.48 | 4.76 | 4.80 | 5.21 | 5.05 | 4.99 |
| IL-5 | BLQ | BLQ |  | BLQ | BLQ | BLQ | BLQ | BLQ | BLQ |
| IL-7 | 61.5 | 9.30 |  | 13.8 | 15.6 | 13.1 | 13.0 | 9.05 | 6.31 |
| IL-8 | 194 | 115 |  | 31.0 | 44.1 | 31.3 | 57.9 | 45.9 | 33.4 |
| IL-9 | 122 | 61.6 |  | 62.2 | 65.0 | 65.8 | 77.5 | 68.4 | 73.6 |
| IL-10 | 20.2 | 14.4 |  | 16.0 | 15.0 | 18.3 | 18.9 | BLQ | BLQ. |
| IL-12(p70) | 68.6 | 42.3 |  | 63.0 | 57.9 | 76.4 | 53.1 | 37.0 | 21.7 |
| IL-13 | 6.49 | 3.80 |  | 4.41 | 5.52 | 9.99 | 10.6 | 6.54 | 2.58 |
| IL-15 | BLQ | BLQ |  | BLQ | BLQ | BLQ | BLQ | BLQ | BLQ |
| Eotaxin | 117 | 61.0 |  | 43.4 | 82.8 | 77.3 | 106 | 148 | 101 |
| FGF basic | 108 | 56.2 |  | 83.3 | 61.9 | 72.4 | 71.7 | 60.4 | 64.1 |
| G-CSF | 44.8 | 51.2 |  | 40.6 | 26.2 | 28.7 | 34.0 | 30.0 | 25.1 |
| GM-CSF | 122 | 23.2 |  | 18.2 | 27.3 | 52.7 | 46.5 | 30.7 | 22.0 |
| IP-10 | 4320 | 1880 |  | 1700 | 1160 | 1110 | 1140 | 1160 | 732 |
| MCP-1 (MCAF) | 91.7 | BLQ. |  | BLQ | BLQ | BLQ | BLQ | BLQ | BLQ |
| MIP-1α | 7.17 | 5.80 |  | 5.57 | 3.82 | 3.65 | 4.26 | 3.35 | 2.19 |
| PDGF-bb | 9220 | 5300 |  | 5720 | 8350 | 7340 | 7330 | 6320 | 5080 |
| MIP-1β | 403 | 161 |  | 76.6 | 93.6 | 111 | 139 | 129 | 98.7 |
| RANTES | 11200 | 9290 |  | 8090 | 10700 | 9780 | 12900 | 11600 | 8480 |
| VEGF | 476 | 219 |  | 234 | 295 | 343 | 276 | 166 | 83.9 |

B

| Day | 87 | 88 | 89 | 91 | 94 | 99 | 127 |
| --- | --- | --- | --- | --- | --- | --- | --- |
| Event | corticosteroid administration |  |  |  |  |  |  |
| IL-1β | 1.87 | 2.02 | 2.00 | 2.00 | 2.06 | 1.91 | 1.89 |
| IL-1RA | 73.7 | 64.9 | 53.0 | 49.8 | 51.4 | 43.9 | 56.2 |
| IL-2 | BLQ | BLQ | 9.52 | 10.7 | BLQ | BLQ | BLQ |
| IL-4 | 5.17 | 4.91 | 5.47 | 5.19 | 5.95 | 5.76 | 5.56 |
| IL-5 | 4.71 | 5.04 | BLQ | 3.29 | 4.36 | 4.36 | 6.18 |
| IL-7 | 7.06 | 6.56 | 7.81 | 6.06 | 6.06 | 5.68 | 8.56 |
| IL-8 | 65.8 | 88.9 | 159 | 72.5 | 88.0 | 41.2 | 76.1 |
| IL-9 | 106 | 112 | 103 | 102 | 108 | 98.8 | 106 |
| IL-10 | 67.8 | 60.1 | 59.4 | 57.9 | 64.7 | 54.1 | 73.1 |
| IL-12(p70) | 45.9 | 52.5 | 39.8 | 39.0 | 50.1 | 47.4 | 77.4 |
| IL-13 | 6.13 | 6.23 | 11.0 | 11.5 | 8.26 | 2.93 | 4.30 |
| IL-15 | BLQ | BLQ | BLQ | BLQ | BLQ | BLQ | BLQ |
| Eotaxin | 73.3 | 51.9 | 45.0 | 46.7 | 79.3 | 91.4 | 60.3 |
| FGF basic | 118 | 116 | 119 | 107 | 117 | 120 | 131 |
| G-CSF | 91.7 | 92.4 | 90.8 | 82.3 | 85.6 | 68.7 | 89.8 |
| GM-CSF | 50.6 | 74.1 | 131 | 88.7 | 60.6 | 25.0 | 46.9 |
| IP-10 | 1670 | 884 | 572 | 617 | 846 | 1620 | 865 |
| MCP-1 (MCAF) | BLQ | BLQ | BLQ | BLQ | BLQ | BLQ | BLQ |
| MIP-1α | 5.31 | 6.06 | 5.02 | 4.89 | 5.69 | 5.69 | 7.06 |
| PDGF-bb | 5360 | 4760 | 6850 | 4700 | 7240 | 6210 | 6270 |
| MIP-1β | 126 | 116 | 100 | 95.7 | 116 | 122 | 106 |
| RANTES | 11700 | 10300 | 13500 | 8270 | 13400 | 14800 | ALQ |
| VEGF | 64.1 | 66.7 | 60.0 | 61.6 | 72.2 | 52.6 | 152 |

ALQ: Above limit of quantification, BLQ: Below limit of quantification ALQ: Above limit of quantification, BLQ: Below limit of quantification
